# Sharing and re-use of phylogenetic trees (and associated data) to facilitate synthesis

**DOI:** 10.1186/1756-0500-5-574

**Published:** 2012-10-22

**Authors:** Arlin Stoltzfus, Brian O'Meara, Jamie Whitacre, Ross Mounce, Emily L Gillespie, Sudhir Kumar, Dan F Rosauer, Rutger A Vos

**Affiliations:** 1 Biochemical Science Division, NIST, 100 Bureau Drive, Gaithersburg, MD, USA; 2 Department of Ecology & Evolutionary Biology, University of Tennessee, 569 Dabney Hall, Knoxville, TN, 37996-1610, USA; 3 NMNH, Smithsonian Institution, Washington, DC, 20013-7012, USA; 4 Department of Biology and Biochemistry, University of Bath, Bath, UK; 5 Department of Biology, Marshall University, Huntington, WV, USA; 6 Center for Evolutionary Medicine and Informatics, Biodesign Institute and School of Life Sciences, Arizona State University, Tempe, AZ, 85287-5301, USA; 7 Department of Ecology and Evolutionary Biology, Yale University, New Haven, CT, USA; 8 NCB Naturalis, Einsteinweg 2, 2333 CC, Leiden, the Netherlands

**Keywords:** Evolution, Phylogeny, Data sharing, Bioinformatics, Phyloinformatics, Standards

## Abstract

**Background:**

Recently, various evolution-related journals adopted policies to encourage or require archiving of phylogenetic trees and associated data. Such attention to practices that promote sharing of data reflects rapidly improving information technology, and rapidly expanding potential to use this technology to aggregate and link data from previously published research. Nevertheless, little is known about current practices, or best practices, for publishing trees and associated data so as to promote re-use.

**Findings:**

Here we summarize results of an ongoing analysis of current practices for archiving phylogenetic trees and associated data, current practices of re-use, and current barriers to re-use. We find that the technical infrastructure is available to support rudimentary archiving, but the frequency of archiving is low. Currently, most phylogenetic knowledge is not easily re-used due to a lack of archiving, lack of awareness of best practices, and lack of community-wide standards for formatting data, naming entities, and annotating data. Most attempts at data re-use seem to end in disappointment. Nevertheless, we find many positive examples of data re-use, particularly those that involve customized species trees generated by grafting to, and pruning from, a much larger tree.

**Conclusions:**

The technologies and practices that facilitate data re-use can catalyze synthetic and integrative research. However, success will require engagement from various stakeholders including individual scientists who produce or consume shareable data, publishers, policy-makers, technology developers and resource-providers. The critical challenges for facilitating re-use of phylogenetic trees and associated data, we suggest, include: a broader commitment to public archiving; more extensive use of globally meaningful identifiers; development of user-friendly technology for annotating, submitting, searching, and retrieving data and their metadata; and development of a minimum reporting standard (MIAPA) indicating which kinds of data and metadata are most important for a re-useable phylogenetic record.

## Findings

Re-use of scientific data underlying published research may take many different forms, including *study replication*, *aggregating* the data with other data of the same type, and *integrating* it with data of other types. In some instances, the form of re-use is unanticipated by the initial researcher (*re-purposing*). Re-use of data is critical to the distinctively self-policing and progressive nature of science, allowing scientists to evaluate and build on the work of others.

Various environmental and technical factors may be assumed to influence sharing and re-use of scientific data: it may be facilitated by software tools and community infrastructure such as public archives; it is guided by institutional policies and informed by educational practices; and it is encouraged (or discouraged) by cultural attitudes. The roles of these factors are apparent in regard to prevailing practices for sharing of DNA and RNA sequence data. Unrestricted sharing was stimulated enormously by journal policies requiring archiving in GenBank [1] as a condition of publication. Software tools and instructions from the resource-provider (e.g., Entrez [2]) make it easy to locate and retrieve archived sequence records. The retrieved records are available in formats readable by many kinds of software, and these records include metadata (e.g., species sources, publication links) vital for interpretation.

The result has been an explosion in scientific productivity in the form of systematic and synthetic research based on re-used sequence data. A similar story could be told in regard to macromolecular structure data in PDB [3]. Note that this explosion in sharing of “data” is not based on the narrow “empirical observation” sense of “data” (i.e., raw data such as sequence traces or crystal diffraction patterns), but implicates synthetic and computed results (1D sequences and 3D structures) crucial to the conclusions of a scientific study.

An explosion in synthetic evolutionary science is also conceivable [4] given similar advances in sharing of comparative data. Evolutionary comparative analysis, which puts comparative data in an evolutionary context, is used throughout biology, in biodiversity studies, systematics, genomics, molecular evolution, and so on. The use of evolutionary comparative analysis is widespread because it represents the appropriate type of statistical analysis to use when comparing entities (e.g., genes, proteins, organisms) that are non-independent samples related by descent-with-modification from common ancestors, i.e., related by evolution. Through comparative evolutionary analysis, biologists infer trees that provide a natural hierarchical classification, and they make functional inferences about molecular, morphological and behavioral traits.

**Figure 1:**
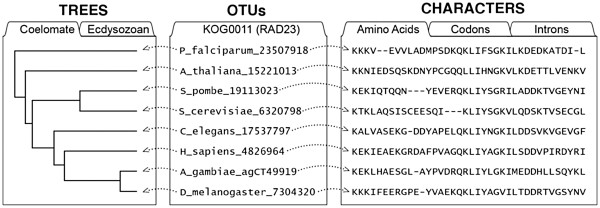
**The character-state data model used in evolutionary comparative analysis.** The character-state data model is illustrated here with an example showing members of a protein family, with a single set of labels for Operational Taxonomic Units, 2 phylogenies, and 3 types of characters (modified from [5]). The biological entities to be compared— whether genes, species, individuals, or some other unit— are known as “OTUs” or “Taxa”. Each OTU may be characterized as having a “state” for a given “character”, e.g., the OTU C_elegans_17537797 has the state “A” (Alanine) for the 2nd amino acid character. Phylogenetic trees (typically, directional, acyclic, singly-linked graphs in which no node has more than one ancestor) connect all the OTUs, representing their descent from a common ancestor.

Comparative evolutionary analysis involves several types of re-useable information, illustrated in Figure 1 (modified from [5]). A phylogenetic tree representing the evolution of a set of entities— called OTUs (Operational Taxonomic Units)— is computed by specialized software, often using an input matrix of “character-state data” consisting of compared traits for the OTUs. Frequently the input matrix is a sequence alignment, i.e., the compared traits (characters) are aligned residues in a sequence, but it may also be a matrix of non-sequence characters, or a mixture of the two. Some comparative studies focus on inferring the correct phylogeny for a set of OTUs, while others focus more on using phylogenetic analysis to test hypotheses or to make inferences about compared traits. 

All of the data from comparative studies are potentially re-usable, from raw observations, to homologized (aligned) characters, to phylogenies and other inferred results. However, in order to be re-used successfully, a scientific result must be stored, discovered, accessed, decoded, interpreted and evaluated— and each of these steps may pose barriers due to lack of the knowledge and the technology that promotes sharing and re-use.

How often are phylogenies (and associated data) re-used? For what purposes are they re-used? Which research areas rely most on re-used data? What are the most important barriers faced by users? The answers to these questions are not known at present. This lack of knowledge makes it difficult for end-users, technology developers, and policy-makers to make the kinds of strategic decisions that would facilitate sharing and re-use of phylogenies and associated data.

To address this deficit of knowledge, we have carried out initial reviews of current practices in publishing and archiving phylogenetic trees; relevant policies of journals and funding agencies; data formats for representing trees and aligned data; and the barriers to re-use experienced by phylogeny users.

We find that re-usable trees are available for only a small fraction of an estimated 7700 studies reporting new phylogenies in 2010: the vast majority of recently published trees are available only as graphic image files, often behind paywalls. Even when re-usable trees are available, they often lack externally meaningful identifiers for OTUs, and nearly always lack methods information sufficient for prospective re-users to evaluate their suitability. Phylogeny-related research depends heavily on the re-use of archived sequences. However, re-use of alignments and trees is increasingly important, particularly the use of extremely large “megatrees” that cover a broad taxonomic group. Scientific users interested in data re-use experience a variety of barriers including lack of archiving, paywalls, scrambled names, untraceable OTUs, incompatible formats, and so on. Current policies are unlikely to alter this situation significantly in the absence of broader community engagement to raise the frequency of archiving, increase the use of machine-processable names, improve the discoverability of archived records, and develop standards and technology to allow the kinds of annotations that users need to evaluate archived results. Nevertheless, the evolutionary research community appears poised to confront these challenges [6].

### Current archiving practices and policies

Our focus in this section is on archiving of data (in public archives and on journal web sites that include supplementary data) as distinct from sharing of data. The major US research funding agencies, NIH and NSF, require sharing of the data necessary to validate a research result (see Table 1 for links to policies). Likewise, funding agencies in other countries (e.g. the NWO in the Netherlands) are starting to require sharing of data. Researchers may be subject to other institutional policies that emphasize the importance of sharing data. Such policies typically urge or demand that researchers maintain good records and make data available upon request— a type of request that, in practice, is subject to delays and (with surprising frequency) refusal [7,8]. An archiving policy, by contrast, specifically requires that data be made accessible in advance via a third-party resource, ideally a public archive [9]. 

**Table 1:** Links to resources mentioned in the text (contact the authors if a resource is no longer available at the given address)

**Name**	**URI or email address**	**Role in sharing of data**
APG tree	http://www.mobot.org/MOBOT/research/APweb/	Authoritative phylogeny from Angiosperm Phylogeny Group
Dryad	http://www.datadryad.org	Public archive of data associated with peer-reviewed bioscience articles
ICBN	http://ibot.sav.sk/icbn/main.htm	International Code of Botanical Nomenclature
ICSP	http://www.the-icsp.org/	International Committee on Systematics of Prokaryotes
ICZN	http://iczn.org/	International Commission on Zoological Nomenclature
JDAP	http://datadryad.org/jdap	Joint Data Archiving Policy that directs authors to submit supporting data to an appropriate public archive
Mesquite	http://www.mesquiteproject.org, http://mesquitelist@mesquiteproject.org	Interactive software for comparative analysis; email list is a common venue for addressing interoperability issues
MIAPA	http://www.evoio.org/wiki/MIAPA, http://miapa-discuss@googlegroups.com	Open project to develop a Minimum Information About a Phylogenetic Analysis standard
MorphoBank	http://www.morphobank.org/	Web tool for sharing and publishing comparative data linked to images and specimen vouchers
NAR database issue	http://www.oxfordjournals.org/nar/database/c	List of secondary resources with alignments and trees (under protein sequences: domain databases)
NESCent	http://www.nescent.org	National Evolutionary Synthesis Center that supports many interoperability projects
NeXML	http://www.nexml.org	Open project to develop an XML format for comparative data and trees
NIH policy	http://grants.nih.gov/grants/policy/data_sharing/	Data sharing policy applicable to NIH-funded research
NSF policy	http://www.nsf.gov/bfa/dias/policy/dmp.jsp	Data sharing policy applicable to NSF-funded research
NWO policy	http://www.nwo.nl/files.nsf/pages/SPES_5VEDDR/$file/Regeling%20subsidieverlening%20NWO.pdf	Data sharing policy applicable to NWO-funded research, policy specified on p19, items 30 and onwards
Phylomatic	http://www.phylodiversity.net/phylomatic/	Software that supports grafting and pruning to create plant phylogenies from APG mega-tree
TDWG	http://www.tdwg.org	Biodiversity information standards organization with an active “Phylogenetic standards” interest group
TimeTree	http://www.timetree.net	Secondary resource synthesizing data on divergence times
iPlant TNRS	http://tnrs.iplantcollaborative.org/	Taxonomic Name Resolution Service for plant names
ToLWeb	http://www.tolweb.org	Secondary resource to assemble a curated tree of life
TreeBASE	http://www.treebase.org	Public archive for published trees and character data.
uBio	http://www.ubio.org	Taxonomic name resolution service for life

In 2011, a group of evolution-related journals announced a Joint Data Archiving Policy (Table 1) requiring data archiving in an “appropriate public archive” to ensure that the data are “preserved and usable for decades in the future” [10]. Some journals have more specific requirements, e.g., the “Journals” page at TreeBASE website (see Table 1) lists 35 journals that recommend or require submission of trees to TreeBASE [11]. Researchers wishing to archive phylogenies or character data in conjunction with a peer-reviewed phylogeny report may use TreeBASE [11], Dryad [9], or MorphoBank [12]. Researchers also may choose to make their data available as supplementary data via a scientific publisher’s web site.

TreeBASE [11] emerged as a project of the systematics community in the 1990’s. As of September 2011, it contained records on 8141 trees from 2864 publications (W. Piel, personal communication). Submission is an interactive, semi-automated process in which the web server imports a character matrix and phylogeny, solicits metadata about the publication, and allows the user to specify an “analysis” link between the tree and the matrix from which it was inferred. Externally meaningful identifiers (e.g. GenBank accession numbers) are not required for submission, but can be added during the submission process. OTU names (in input files) that follow the pattern < genus > <species > <other_qualifiers > will be detected and parsed to yield a user-approvable link to the identifiers used by 2 major online resources for taxonomic identifiers (UBio and NCBI). In practice, the need for an input file in a compatible NEXUS format [13] has been a significant hurdle for some users, though knowledgeable users may create compatible files with tools such as Mesquite [14] following video instructions on the TreeBASE website.

Dryad [9] began in 2009 and has been designed with a larger community in mind, being governed by a consortium of journals. Like TreeBASE, Dryad is an archive for publication-associated data. Unlike TreeBASE, it does not restrict users with respect to formats or data types, but encourages users to rely on simple, portable formats, and to adhere to any relevant community standards. Unfortunately, there is no accepted community standard for a phylogenetic report, notwithstanding recent efforts in regard to a MIAPA standard [15] described below. An indication of this deficit is that most Dryad packages for phylogenetic reports in the 2010 publication year actually do not contain a phylogeny in decodable form (see Supporting Data).

MorphoBank [12] is designed to support collaborative sharing and archiving of comparative morphological data, as opposed to trees, on the premise that much of the re-useable information in a comparative analysis of morphology is not in the published tree, but in the character matrix, and particularly in the specimen identifiers and photographic images linked to character-state encodings. MorphoBank also allows molecular characters, as these often are mixed together in phylogenetic analyses (e.g., study #563). As with TreeBASE and Dryad, a private record can be created and revised prior to making it public. Indeed, the design of MorphoBank makes it highly useful for pre-publication sharing of data among collaborators, and as a result, there are more private projects in MorphoBank (440) than public ones (154).

How often do researchers deposit phylogenetic trees and associated data in a public archive? We estimate the frequency of archiving for the publication year 2010, using the number of archived phylogenetic studies, and an estimate of the number of publications reporting phylogenies. As of August 2011, 307 studies with publication dates in 2010 have decodable phylogenies archived in TreeBASE (Bill Piel, pers. comm.) or Dryad (Supporting Data), the vast majority (300 studies) in TreeBASE.

To estimate the total number of phylogeny reports in 2010, we searched the expanded citation index of Web of Science (Thomson Reuters, 2011, http://www.wokinfo.com) for entries with 2010 publication dates that matched “phylogen*” in any field, finding 11,664 records (see Supporting Data for details). This number may be an over-estimate due to publications that refer to a phylogenetic concept but do not report a new tree. To estimate the rate of such false positives, we chose a random sample of 100 publications for direct examination: 66 actually reported a new phylogeny. False negatives in the form of phylogeny-relevant articles that match “tree” (or “cladogram”, “dendrogram”, etc.) without matching “phylogen*” are rare: we estimate them at < 1 % of the “phylogen*” records. Thus, on a per-publication basis, the frequency of archiving in a public archive is 307 / (0.66 * 11664) = 4.0 %, or about 1 in 25. This corresponds rather precisely to a somewhat narrower estimate by Hughes [16], who tested optical tree-recognition software on images downloaded from 249 articles published in BMC Evolutionary Biology (an open access journal) from 1997 to 2009, noting that archiving of alignments and trees in TreeBASE— which obviates the need for optical tree-recognition— occurred in just 11 cases, i.e., 11/249 = 4.4 %.

What about journal web sites? To assess the extent of archiving via journal web sites, we examined 40 recent articles from the top of the list (ranked by relevance) of articles in Web of Science that matched “phylogen*” in the title or topic (described further in the section below on re-use; see Supporting Data). Most articles, along with desired supplementary data, were obtained from the publisher, often via an institutional subscription, and the remainder were obtained from the authors in response to a personal request. We note in passing that journal publishers often place supplementary data behind a paywall (e.g., in [17-19]). Of these articles, 38 presented a new phylogeny, and 34 presented new homologized characters.

**Figure 2:**
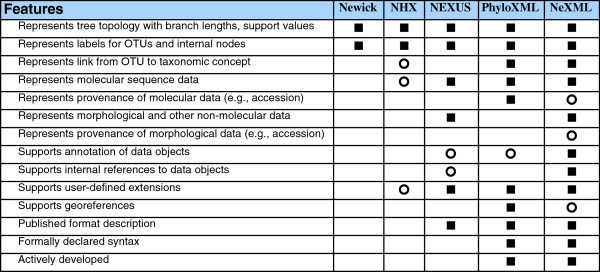
**Comparison of file formats commonly used to represent trees.** The features of various formats in common use are compared, with a square indicating support for a feature, and an open circle indicating partial or incomplete support. The Newick format represents trees (and no other information) as a series of parenthetical statements representing internal nodes, taxon names, and optionally branch lengths (as described in http://evolution.genetics.washington.edu/phylip/newicktree.html). NEXUS [13] utilizes Newick strings, but also may store character information, processing commands (e.g., to exclude certain OTUs or characters), and notes. There is no formal way to propose extensions to NEXUS, but it has been widely adopted. PhyloXML [20] can store trees and molecular data, as well as accession numbers, geographic information, and other data. NeXML [21] is a different data format intended as an XML-based replacement for NEXUS. Both PhyloXML and NeXML have a formal syntax in an XSD schema. For further information, see [21].

Phylogenetic relationships— including topology, branch lengths, and support values— may be encoded in various common formats [13,20,21] whose features are compared in Figure 2. We found 2 cases in which decodable phylogenies in Newick (nested parentheses) format were provided, though in minimalistic form, without branch lengths or support values. In one case, a table in the main text compares support for various trees represented symbolically, albeit OTU names are highly abbreviated in order to condense the tree-strings to fit the table [22]. In another case, tree-strings are given in an appendix [23]. 

Thus, the frequency of archiving decodable trees on journal web sites is 2 out of 38. In addition, in this same set of 38 articles, we identified 2 cases of archiving in TreeBASE [24] or Dryad [25]. Thus, in this sample, the total frequency of archiving decodable trees is 2/38 for public archives (similar to the frequency seen in the 2010 sample above), and 2/38 for abbreviated Newick strings in journal-associated content. This represents the state of archiving before the Joint Data Archiving Policy went into effect [10].

Interestingly, half the articles that present new phylogenies provide, as supplementary data, images of additional trees not appearing in the article. While such images may assist readers in judging scientific claims, they do not favor re-use, relative to sharing the logically encoded tree (typically a Newick file) that the authors must have used to construct the image. Just as typical word-processing software does not accept pictures of text as inputs, and mathematical tools do not accept pictures of equations as inputs, typical phylogeny-related software for viewing, manipulating or analyzing trees (e.g., RAxML, PAUP*, Archaeopteryx) does not accept pictures of trees as inputs: tree-pictures are outputs, not inputs, to analysis tools. Software exists to assist users in reconstructing a tree from a tree image, but even the best available tool for optical recognition of trees [16] has a high failure rate (only a minority of trees are rendered in the right shape to allow processing) and does not even attempt to recover support values (e.g., bootstrap values). No matter how good the algorithm, the strategy of re-using phylogenetic information via optical tree recognition suffers from the same flaws as the strategy of transferring textual information by printing an electronic text file on paper, taking a photo of it, and then using optical character recognition to decipher the image and store the results as electronic text. By contrast, trees represented logically and encoded as text in an electronic file (e.g., Newick, NEXUS, NeXML, or PhyloXML) can be decoded without loss of information on topology, branch lengths, OTU labels, and support values.

By contrast to the case for trees, we found many positive examples of archiving other types of data. With respect to unaligned data, the public archiving of sequence data in GenBank is very high (consistent with [26]). For non-sequence data, archiving is atypical, though it occurs, e.g., measurements of fungal oogonia in [18] appear in a supplementary table. With respect to aligned (homologized) data, we found many examples of authors making data available as online supplements: pollution tolerance measures in chironimids and mayflies [27], measurements of virulence and other factors in *E. coli* strains [28], wood traits and collection data [29]. The study of wood traits [29] also provides an example of how inferred ancestral trait values (which cannot be represented in common data interchange formats used in phylogenetics) may be conveyed in tabular form, with nodes indicated by taxonomic splits (e.g., "Gymnosperms versus Angiosperms").

### Current practices of re-use

What does scientific re-use of data look like? How often does research depend on the re-use of phylogenies and associated data? Does re-use focus on trees, alignments, unaligned characters, or other information? How often does re-use take the form of systematic aggregation from many resources? Here we draw a crude picture of phylogeny-related data re-use based on (1) the previously mentioned systematic examination of a sample of 40 recent high-relevance phylogeny-related articles, supplemented with (2) a superficial survey of all articles (whether phylogenetic or not) in the April, 2011 issues of two specialized journals, Evolution, which features evolutionary studies, and American Journal of Botany, which frequently features phylogenetic studies, and (3) other published studies familiar to the authors.

#### Sequences and other unaligned characters

Sequences represent the type of data most commonly re-used in phylogeny-related studies, being seen in just over half the cases (21 out of 40) in our random sample of high-relevance articles. Nearly all phylogenetic studies that use sequences rely on pre-existing sequences; rarely, a phylogenetic study relies solely on newly determined sequences, as in [30]. The most commonly indicated source of sequences is GenBank [1]. However, one publication [19] indicated the Barcode of Life Data (BOLD) system [31] as the source of some sequences.

In regard to re-use of non-sequence data, we encountered two studies that aggregated large amounts of data from other publications and used these in some type of phylogenetic analysis, one of them addressing wood traits [29], and the other exploring allometry (i.e., relationships of scaling) in regard to milk intake in mammals [32]. In both cases, the data were provided in the form of tables in the publication or its supplementary data.

In addition to these isolated cases from our literature sample, there are hundreds of secondary resources devoted to the re-use of sequence data, including dozens of databases that assign sequences to family clusters (see Table 1, NAR database list), often providing alignments and even trees, e.g., Pandit [33], Pfam [34], or COG [35].

#### Aligned (homologized) characters

When re-use of aligned (homologized) characters occurs, it is most often that the authors are adding to their own previous work, i.e., the authors add new rows or columns to an alignment from a study with an overlapping set of authors (e.g., [36]). It may seem surprising that authors do not simply re-align all the data, but in many studies, authors are using manual methods of alignment, either with non-sequence characters for which there is no automated method (e.g., [37,38]), or with sequences so closely related that manual alignment is not problematic (e.g., [39,40]).

In 2 studies from the random sample of 40, authors relied on a secondary resource for homologized characters. One study [41] used BaliBASE, a benchmark alignment database, to understand how multiple alignment affects phylogeny inference, and another study [42] used several resources (COG, Tribes, and OFAM) to assess how orthology assignment affects phylogeny inference.

While sequence alignments are readily available in secondary resources noted above, morphological and physiological characters are harder to find, and seem to be valued more highly. The leaf functional traits data in [43], re-used by Walls [44] in our sample of 40 articles, would appear to be enormously valuable. To assess how frequently these data have been re-used, we examined 40 randomly chosen articles that cite [43], finding that 8 of them (20 %) represent cases of data re-use, implying an expectation of 188 cases among the 940 papers that cite [43].

#### Phylogenies

Surprisingly, in the sample of 40 recent phylogenetic articles (see Supporting Data for details), we found that 5 studies rely on the same suite of phylogeny resources, namely the phylogeny of plant taxa maintained by the Angiosperm Phylogeny Group (APG; see Table 1) and available via Phylomatic [45]. In four of these studies, the APG tree is used as the main basis of phylogenetic analysis of biological data, sometimes by refining or extending the tree: Duarte [46] uses the APG tree to measure the phylogenetic diversity of species assemblages found in different forest patches; Zhang, et al. [29] use the APG tree as the backbone for a supertree used to analyze wood traits in 608 species; Walls [44] uses different versions of the APG tree (and the tree from [47]) in an analysis of leaf vein patterns; in an analysis of scaling relationships in phylogenetic diversity, Morlon, et al. [23] heavily supplement the APG backbone with other phylogenetic results. A fifth study [48] uses the APG tree from Phylomatic as a standard of comparison to validate its own tree. These examples reflect the ready availability of a mega-tree covering plants. A comparable resource covering animals would be the supertree of mammals in [49], which is used once in the set of 40 articles, namely Riek’s analysis of milk traits [32].

Phylomatic [45], employed in several of these studies, is itself an example of re-use. The APG periodically develops a consensus view of angiosperm classification based on phylogenetic information. This is combined with other information from phylogenetic studies to create a megatree that is available via an installable software package called Phylocom, which includes the Phylomatic application. A comparable resource with a different focus is the Tree of Life Web Project (ToLWeb) [50], which consists of curated pages with phylogenies and additional data for various groups. ToLWeb seems to be used mainly for educational purposes, rather than research. The NCBI taxonomy hierarchy [1], though not strictly a phylogeny of life, is widely used as such, e.g., in TimeTree [51] and various other projects [52-61].

The re-use of more narrowly defined species trees, or of gene trees, is less common, though examples may be found. For instance, Wright [62] analyzes the evolution of mimicry in a group of rift lake catfish, using a species tree for this group that was generated two years earlier by others [63]. In regard to gene trees, the example of [64] indicates that a species trees may be inferred from a set of trees from many different gene families (nearly 19 000 in this case), nevertheless, this is not an example of data sharing, because the input trees were generated in the same study.

### Current barriers to re-use

**Figure 3:**
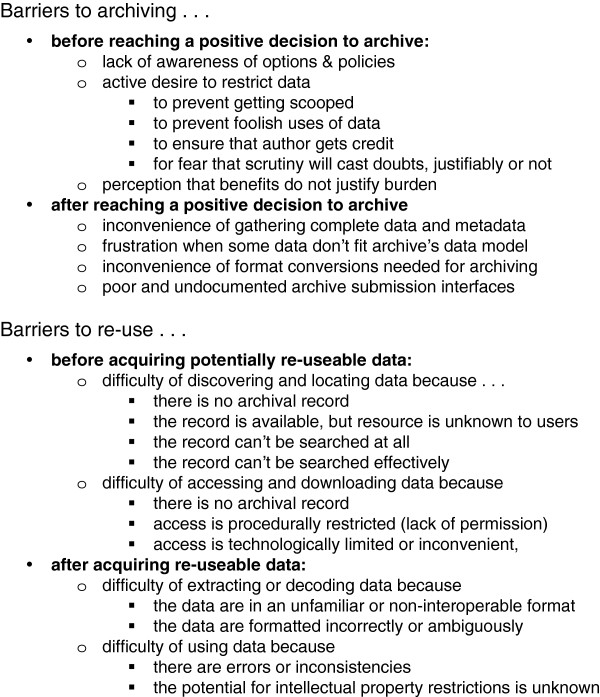
**A taxonomy of barriers experienced by users.** Barriers may occur at many different steps along the path of re-use. For instance, an author may decide not to archive data, due to the perceived burden. If the author does not archive data, then it is difficult for users to discover that the data exist. Once the user discovers that the data exist by reading a publication, the only way to obtain the data is to write to the author, a process that is known to be subject to delays and refusals. Even if the data are placed in an archive, it may be difficult for users to discover (e.g., journal web sites typically do not offer any kind of content searching for supplementary data) or to access (e.g., users may be required to pay for access). Finally, it is not unusual for archived data to contain errors and ambiguities that make it difficult to apply in scientific research.

As indicated in the taxonomy of barriers in Figure 3, conditions that inhibit the re-use of data might occur at many points, from a producer’s initial decision not to archive data, to a re-user’s final decision not to incorporate (in a published study) data that were archived, discovered, acquired, decoded, and evaluated. For the present purposes, we discuss barriers tentatively, with no intention of being complete or systematic. We draw on our own experiences and those of others: the authors are evolutionary researchers who have carried out phylogeny-based research and have experience with data re-use; as part of our study, we contacted other researchers (listed in Acknowledgements) to discuss their experiences (see the “user stories” in Supporting Data).

#### Barriers to discovering relevant data

Users interested in phylogenies frequently report the impression that re-usable data to match their needs do not exist. For instance, the authors of a study to determine how the invasiveness of a species depends on the relatedness of native competitors constructed a new phylogeny, explaining that they “could not find a reptile phylogeny spanning the breadth of reptile taxa native and introduced to California and Florida” [65]. Clearly, these needs are very specific: the vast majority of published phylogenies will not suffice, because they do not cover the case of interest to the users. The narrowness and specificity of users’ needs is not itself a barrier to re-use, but suggests the importance of discoverability.

Barriers to discovery become more obvious if we compare the re-use of trees with the re-use of sequence data. In the case of sequences, users may assume that > 90 % of published DNA sequences are archived [26] in a single resource, GenBank [1]. The records in this resource can be discovered by a variety of means, including by reference (e.g., an accession number), by text-based searches of metadata, by links from a publication database (PubMed), by a taxonomic hierarchy, and by content-based analytics (similarity searches). Users are free to download and use the discovered records. In addition, GenBank has programmable interfaces, including a web-services interface, allowing users to write programs that carry out automated search and retrieval tasks.

The situation in regard to phylogenies is much more complex and difficult. Even if an earlier phylogeny exists, it probably has not been archived (due to the low frequency of archiving noted earlier). Even if it has been archived, it may remain undiscovered in the absence of a comprehensive resource (of all archived trees) that is well known to users, and that provides powerful search interfaces. Indeed, among the resources that provide phylogeny-relevant data, we know of none that would support the type of query demanded in the case noted above, i.e., to search for any available sequence alignment or phylogeny with a set of OTUs such that the OTUs (1) are in a given taxon (reptiles) and (2) include both positive and negative values of a given ecological trait (invasiveness) and (3) have been collected in one of two given locations (Florida or California). TreeBASE supports (1) taxonomic searches, but not the other two search criteria. Journal web sites typically do not support any queries of supplementary data records: they are discoverable only by reading the article or visually scanning its web page for the presence of “Supplementary data” links.

Nevertheless there are highly usable secondary archives that provide access to large numbers of alignments or trees (see Table 1, NAR database issue). Our experience responding to requests from colleagues for help with phylogenetics is that users frequently are unaware of how to use such resources effectively.

Given that the frequency of archiving is low, discoverability of archived resources is low, and awareness of primary and secondary resources is low, the question arises, how do users search for data that meet their needs, before concluding that no such data exist? In practice, the approach favored by researchers seems to be either (1) person-based, via person-to-person networking or keyword searches on the web to identify experts, or (2) publication-based, starting with a keyword search in a reference database such as Web of Science or PubMed. Both approaches converge at the point where the user identifies a potentially relevant publication. The publication is studied, and the author is contacted with further questions and possibly a request for data. The fact that publications (and publication metadata in literature databases) are central to the discovery of supplementary data suggests ways that resource-providers can improve searching (see recommendations below).

#### Barriers to accessing, decoding and evaluating candidate records

Once a record (or a resource) with potentially relevant data is discovered, the prospects for re-use depend on accessing (or extracting) and decoding the data, and evaluating its suitability. Access barriers can occur regardless of where data are being stored. Re-use frequently is mediated by direct user-to-author communication, rather than by an archive or a journal web site. Scientists who request data from authors often face delays and refusals [7,8], resulting in an access barrier. Anderson [66] showed that, in a minor but substantial fraction of cases, supplementary data stored on web sites, including some journal web sites, quickly becomes unavailable. In addition, access to supplementary data stored by a scientific publisher may be restricted to paid subscribers.

The inability to decode data in a recovered resource might seem rare, but we have mentioned it already in regard to trees. A phylogenetic tree published as a graphic image— whether embedded in a journal article or provided separately as a supplement— is largely an informational dead-end. We say “largely” because software exists to reconstruct a symbolic representation of a phylogeny from a figure [16,67,68]. The existence of 3 different implementations of this concept suggests a demand for re-usable trees. The same demand for re-usable data is indicated when users go to the trouble of hand-entering a matrix of morphological character-state data from a printed publication, a situation described twice in our user stories (see Supporting Data).

Even when potentially usable data are discovered, accessed and decoded, there remain barriers to their ultimate use. One colleague states (see Supporting Data):

“I have often tried to reuse or reference phylogenies reported by other researchers . . . the biggest hurdle is usually acquisition of the actual sequences used for those phylogenies. Most frustrating are phylogenies in which the sequences used are given generic names (which basically makes it impossible to replicate). Obviously, such cases make it impossible to reuse the phylogeny. In cases where I have succeeded in reusing a reported phylogeny, it has involved repeating the analysis from alignment through phylogenetic analysis.”

One barrier suggested by this comment is the use of entity names that have meaning to the original author, but no external meaning. Deciphering the names may require reading the original publication or contacting the original author. Another recurrent problem is the use of inconsistent naming schemes, i.e., cases in which different names for the same entity are encountered, within the same file or data package.

Perhaps the final barrier, after discovering, accessing, and decoding re-usable data, is the difficulty of evaluating the robustness and stability of the result (i.e., assessing quality). As indicated in the above quotation (and in a case noted earlier in regard to [62]), users may address a concern about quality by attempting to replicate the previous study. Yet replicating a study may be impossible due to incomplete or inaccurate description of methods. Such replication to evaluate quality would not be necessary if the user had reliable indications that a result is of low or high quality. In the case of alignments and trees, for which the external standard of truth— actual evolutionary history— is inaccessible, quality seems to be judged mainly by whether the methods of computation are perceived to have been chosen with accuracy (as opposed to ease-of-use or computation speed) in mind.

### Recommended practices and strategic opportunities

A major impetus for this analysis was to provide a foundation of knowledge for making strategic decisions, so that resources can be allocated in ways that are likely to produce benefits for the community of scientists carrying out evolutionary research. Here we provide suggestions for facilitating the re-use of phylogenies and associated data, considering the differing perspectives of data producers, secondary consumers, resource-providers, policy-makers, and the research community as a whole.

#### Producers of shareable data

Producers of potentially re-usable data can facilitate data-sharing by making a positive decision to preserve data in a public archive; by choosing to provide data in an easily decodable form; by assigning externally meaningful identifiers to data entities; and by annotating results with appropriate metadata.

The first obvious suggestion for producers of data is to make a positive decision to archive data. Reaching a positive decision may be easier if one is aware that offering open access to data in a public archive benefits the scientific community as well as the individual researcher, via increased citations [69]. Another motivation for deciding to archive is that funding agencies increasingly require this.

Where should results be archived? For users aiming to ensure that their data will remain intact, accessible and discoverable in perpetuity, public archives are preferable, while journal web sites are a poor choice. Journal web sites (1) fail to provide search interfaces that make supplementary data easily discoverable, (2) often require payments to access the data, (3) are not committed to ensuring the long-term preservation of the data, and (4) leave users confused about potential copyright restrictions on use of the data. None of these conditions apply to public archives such as Dryad and TreeBASE.

Second, data for archiving should be encoded in non-proprietary formats from which data can be extracted without loss, which for practical purposes means using text-based formats, not graphic images files or binary files. For phylogenies, various text formats are in common use (Figure 2). Most types of character data can be represented in NEXUS [13]; there are also various formats designed for molecular sequence alignments (e.g., FASTA, PHYLIP, MSF, ClustalW, MEGA, PIR). For other tabular data, comma-separated value (csv) files are highly portable, and are supported for import or export by popular spreadsheet software such as Microsoft Excel. The custom— encouraged by journal web sites— of publishing tables of supplementary data as PDF documents does little to facilitate re-use. PDF is designed to be a portable document standard for human-readable documents, but it is not designed for portable data management, a fact that becomes obvious if one frequently has the need to extract usable data from a table in a PDF document (e.g., Table 1 in [32]).

Third, it is important to use consistent OTU names (and more generally, entity names) that are externally meaningful. The tree “((A, (B, C)), (D, E))” has no information value unless one knows what are the entities designated by A through E. A phylogenetic tree is useful only to the extent that its nodes are not merely anonymous nodes, but can be linked to data and metadata, in the same study or in other studies. In other words, the re-use value of a phylogenetic result depends on the identifiability— ideally, by a computer, without human intervention— of the OTUs.

Thus, OTUs should be given externally meaningful identifiers, e.g., a recognized species name (or taxon identifier from NCBI), an LSID, a museum specimen identifier, a GenBank accession number. The identifiers could be assigned directly, but given that authors seem to prefer customized, human-readable names with a local meaning, other approaches are to use semantic tagging [70,71] or to provide a separate mapping (e.g., in the form of a simple text table) between local names and externally meaningful identifiers. This is especially important, and especially feasible, when the OTUs represent species, as species names are probably the most important non-molecular means of aggregating and integrating biological data [6,72,73]. This raises the question of which species names to use. Ideally, recommendations from recognized biological codes of nomenclature such as ICZN, ICSP or ICBN (see Table 1) will be followed. Given the continuing failure of the research community (other than in the case of prokaryotes and, soon, fungi [74]) to maintain official, required registration of names, there has been a proliferation of partially-conflicting name databases to fill this need (IPNI, Tropicos, Global Compositae Checklist, Index Fungorum, and many more). Thus, authors should indicate which taxonomy they are using.

Finally, the value of an archival record is greatly enhanced by annotations that make the data more intelligible for secondary uses. Some data formats (NEXUS, NeXML, PhyloXML) allow for some metadata to be included in the same file as the data, while in other cases, metadata can be provided in a separate file. Precisely what should be included? The rather ambitious “minimum” list provided by Leebens-Mack, et al. [15] specifies: “(1) a description of the objectives of the phylogenetic analysis and the component trees included in a study …; (2) the raw sequences or character descriptions; (3) sample voucher information; (4) a description of procedures for establishing character homology (e.g., sequence alignment); (5) the sequence alignment or some other character matrix; (6) detailed description of the phylogenetic analysis, including search strategies and parameter values (specific commands for the analysis program would be optimal); and (7) the phylogenies including branch lengths and support values (e.g., bootstrap)”. A more precise MIAPA checklist recently emerged from a workshop staged by the TDWG Phylogenetic Standards Working Group (see Table 1 for MIAPA and TDWG resources). The value of such a checklist will depend on software that makes it easy to use.

#### Consumers of shareable data

The consumers or “re-users” of data typically are producers in the sense of generating synthetic or value-added results— they can facilitate sharing of these results by following all of the suggestions above for producers of shareable data. Consumers also have a unique responsibility to cite published reports of re-used data, as part of the social contract of science.

However, because published papers rarely contain complete data in usable form, fulfilling the obligation to cite prior literature is not the same as fulfilling the obligation to provide provenance, i.e., to explain the source of data. In order to clarify this point, we provide some examples. For instance, Zhang, et al. [29] present data on 11 wood traits for 608 species, saying that these "were compiled from the literature" and citing 3 sources; Riek [32] states that data on 8 lactation traits for 40 mammal species "were obtained from the literature", citing 45 sources in an elaborate table of data; Bjarnason, et al. [75] cite 3 publications as sources of morphometric data; Morlon, et al. [23] provide an appendix with phylogenies (in Newick format) attributed primarily to Forest, et al. [76]. In all cases, the authors have fulfilled their obligation to cite published literature.

However, while these authors have followed prevailing standards and are not guilty of any oversight, the actual source of data, and the path they have taken, is unclear in every case. Forest, et al., the source cited by Morlon, et al. for phylogenetic relationships of over 600 plant genera, present only images of phylogenies, in supplementary data files. Possibly Morlon, et al. recovered information by optical tree recognition, but more likely, the phylogenetic information was obtained as an electronic file communicated personally by Forest, et al. The 3 sources of morphometric data cited by Bjarnason, Chamberlain and Lockwood [75] comprise 1 article by Chamberlain and 2 by Lockwood, et al.: when authors attribute data to their own prior studies, the continuity of data probably reflects an electronic file that one of the authors has stored privately on a computer. As Riek’s data came from many sources, each with only a few data values, it is likely that Riek read each paper and used manual keyboard entry to record the values in an electronic file. Zhang, et al. may have done the same, but this seems unlikely, as the data in their matrix of 6688 values are attributed to exactly 3 books. Possibly they parsed the data from electronic copies of the books, or used optical character recognition to process large tables of data from the printed books.

In most such cases, a single carefully worded sentence would suffice to make clear to the reader precisely how an electronically encoded version of the data came to be in the possession of the authors. Such a sentence might begin with the words “We obtained an electronically encoded version of the data in < *specific page, table orfigure number* > of < *published work*>” and would continue either with “from < *person* > on < *date* > (personal communication)” or else with “by {keying in values manually | copying and pasting values | image-processing using < *optical recognition software*>} from < *specify paper or electronicsource*>”.

In the case in which data are obtained from a web site associated with a database or archive, the authors should cite a database publication where available, and provide, whenever possible, an identifier for the electronic record that is unique for that archive or database (e.g., accession number). If no such identifier exists, the author may provide a URI, although this is a risky strategy. URIs are volatile, and it is easy to make mistakes in recording them (e.g., the phylogeny URI in [46] or the BAliBASE URI in [41]).

#### Journal editor, publishers, and policy-makers

Although journal web sites are not the preferred venues for archiving when a public archive is available, enforcement of archiving policies by journals has had an enormously positive impact on the public availability of DNA sequences and of protein structures. The prospect of publication is a major inducement to authors, representing an opportunity to compel authors to honor the “social contract” [9] to share data.

Journals can improve the opportunities for sharing of data by taking steps to ensure that data are accessible and discoverable. Some steps are simple and could be undertaken by a journal with little effort. We have mentioned already that journals typically offer no search interfaces to supplementary data, a situation that is easily remedied. Journals also may wish to consider simple ways to expose the fact that an article is associated with supplementary data or an archival record. Some journals (e.g., Bioinformatics) already include, within the published abstract, a “Supplementary information” subheading. When such information, including identifiers, is added to an abstract, it enters the stream of syndicated metadata already provided by publishers, and thus becomes discoverable using resources such as PubMed.

With greater planning and coordination, publishers could implement a more effective system. Scientific publishers already recognize that it is in their best interest to syndicate metadata about scientific articles (authors, title, citation, abstract, keywords), so that secondary resources (e.g., PubMed) can provide aggregated metadata to users, increasing the discoverability of the articles. The same approach can be used to improve the discoverability of supplementary data records associated with a publication. Each such supplement first must be treated as a unit of content with a stable identifier (ideally a DOI or other globally unique identifier). Then the record can be made discoverable via an automatic service that associates this identifier with its publication, whether the identifier is for a record on the journal’s web site, or in a public archive. Such a linking service will make the archival record automatically discoverable via the publication record, whereas currently one has to examine (by eye) a printed article or its journal web page to discover if supplementary data are available.

Finally, given that scientific journals have embraced the role of enforcing policies about archiving and sharing, it is in their interest to make compliance and enforcement as easy as possible for authors and reviewers. Two non-technological ways to do this are for journal publishers (or the professional societies that contract them) to (1) invite articles providing discipline-specific instructions on best practices; or (2) provide a forum for associate editors and authors to share knowledge of best practices.

#### Infrastructure developers and resource providers

The research community includes a small but potentially influential subset of technologists who devote efforts to developing open-source software tools and infrastructure useful to other scientists. These resource-providers and infrastructure-developers can facilitate sharing in a variety of ways: producing “how to” documents, providing better infrastructure to support common re-use cases, supporting annotation through development of tools and vocabularies, and providing name-resolution services.

Perhaps the most obviously remediable barrier to data re-use is the general ignorance (noted earlier) of the practices that facilitate sharing, and of the tools that support interoperability, e.g., producers of phylogenies frequently choose to archive images of trees, even though nearly all tree-rendering tools use the familiar Newick input format, which would make trees shareable without loss of information. Likewise, we have heard several indirect complaints about the TreeBASE submission process, even though its requirement for a NEXUS file is (in most cases) relatively easy to satisfy using an interactive program called Mesquite [14]. Users seem to be unaware of numerous resources that provide pre-computed phylogenies and trees; and unaware of the ease of some obvious modes of incremental re-use, such as adding a new sequence to an existing alignment (which can be done by ClustalW, for example).

Because the technologists in the evolutionary research community have the most knowledge of standards and of tools available to users, they could have a major impact on re-use simply by developing and disseminating how-to documents, tutorials and other information resources. Various topics could be addressed: translating among various alignment and phylogeny formats; instructions for documenting a standard phylogenetic analysis according to the MIAPA checklist mentioned earlier; ways to make use of online databases of sequence families, alignments, and trees.

A second way that infrastructure developers and resource providers could facilitate re-use is to focus on the forms of re-use that are of greatest interest to users. Earlier it was noted that 5 studies in an apparently random sample of 40 recent high-relevance papers all used the APG megatree as an input. This high frequency surely does not reflect an unusually high level of end-user interest in the phylogeny of plants (as opposed to other organisms), as much as it reflects the combination of (1) a phylogeny that provides enormous coverage of a group of organisms (the APG tree) and (2) a software tool (Phylomatic) that makes its use convenient (there may be additional factors: the APG tree may be a better or more authoritative tree; or it simply may be better known among researchers). Surely this success could be replicated in the other kingdoms of life (animals, fungi, protists and prokaryotes).

An additional need is for tools to annotate phylogenetic records. Perhaps the best current example of such a tool is the submission interface for TreeBASE, which enables users to carry out some essential tasks, such as linking OTU names to species names, and annotating the methodological link between a tree and the data matrix from which it was derived. The results of this process are incorporated into TreeBASE’s internal database. Satisfying a more general need for archive-ready records would require a tool that, separately from submission to TreeBASE (or any other archive), generates an output file that meets a minimum standard of annotation (see comments on MIAPA below). This could be a stand-alone tool, or it could be integrated into the software used by researchers to generate phylogenies (most phylogenies are generated by a small set of popular software packages). NeXML, a recently developed file format for comparative data and trees [21], would represent a suitable output format for this type of annotation system (see Figure 2).

The success of annotation tools presumably will depend on the development of controlled vocabularies for annotation. The extensibility of NeXML, for instance, depends on its ability to draw from external vocabularies, which means that the external vocabularies need to exist first, e.g., in order to annotate the methods used to generate a result, one needs a vocabulary of methods. At the very least, the vocabulary will establish relationships of equivalence (synonyms), so that when (for example) one searches a database for all trees generated by the program PAUP*, one does not have to enumerate all possible variants of the name (PAUP, PAUP*, Phylogenetic Analysis Using Parsimony, PAUP4.0b10, and so forth), nor exclude other programs that might give a false match (PaupUp, PAUPRat, etc.). In addition, if the vocabulary for annotating methods is structured (as a taxonomy, or as an ontology), then it will be possible to make logical inferences about the methods used in a set of records, e.g., it will be possible to search for all trees inferred using parsimony, excluding those inferred using likelihood or other criteria.

Finally, the importance of comprehensive technical support for assigning and decoding distinctive identifiers cannot be over-emphasized. Integrating data of different types requires an integrating variable, which in biology is often the proper name of a biological entity [72,73]. Data in publications, supplementary data files, and archival records are only useful to the extent that the entities have externally meaningful identifiers— identifiers that other scientists can decipher. Linking each entity to a particular species using a globally unique identifier, such as a life science identifier (LSID; [77]), NCBI taxon identifier, or another identifier, would greatly facilitate reuse. Thus, one aspect of support for naming is simply to provide users with interfaces that allow assignment of externally meaningful names. This entails avoiding arbitrary limits on name lengths, which entails either avoiding the software and file formats that place such arbitrary limits, or wrapping them in other software that interconverts full names and abbreviated names.

Until use of such identifiers becomes commonplace, and perhaps even after it becomes commonplace, the problem of identifying and matching names will remain a significant barrier [72,73,77]. Some taxonomic name mismatches are unavoidable due to changes in nomenclature. Most other mismatches reflect the desire for human-readable, contextualized names, rather than computer-readable globally unique identifiers. For example, one phylogenetic study on the beetle group *Sitophilus* might list a particular taxon as *S. oryzae*; a broader study might list the same entity as *Sitophilus oryzae*; and a third study might list a GenBank accession number for a sequence, AY131070, without an explicit species name. The prospects for sharing of data will depend considerably on practical automated approaches to reconciling names, as in the Taxonomic Name Resolution Service offered for plants (see Table 1). For widespread automated sharing of data, this kind of service needs to cover the entire tree of life. Taxonomic name resolution remains a difficult problem, e.g., given that “*S. oryzae*” might refer to the beetle *Sitophilus oryzae* in one study, and to the fungus *Sacrocladium oryzae* in another study, names cannot be fully resolved without mining the context for clues.

#### Community

The future prospects for developing a rich economy of shareable data and synthesizable results in phylogenetics will depend on development and implementation of standards that favor re-use of the published information. However, no formal reporting standard exists for a phylogenetic analysis in spite of an articulated need for a Minimum Information About a Phylogenetic Analysis (MIAPA) [15].

How would such a standard develop? Some standards are *de facto* standards that emerge without a deliberate community-wide process. The emergence of a *de facto* standard sometimes reflects market forces, as in the history of formats for music and video recordings. The Newick (New Hampshire) and NEXUS [13] standards originated when small groups of phylogenetic software developers hashed out a common form for representing data. Over the years, these formats became standards because they were adopted by other software developers and continued to satisfy the needs of users.

In other cases, development of standards follows a more deliberate community process, e.g., the development of a next-generation HTML standard (HTML5) by the World Wide Web Consortium (W3C). Such processes typically have the goal of satisfying the entire community of stakeholders affected by the prospective standard; they begin with a working group that produces a draft, and proceed through internal reviews, public RFCs (requests for comment), and revisions.

Thus, whether deliberately or not, the emergence of standards is a community process. To ensure that their needs are addressed in this process, end-users, developers, and policy-makers should be aware of opportunities to participate in organizations and projects that are active in developing standards or supportive technologies, some of which are listed in Table 1. The Biodiversity Information Standards organization (TDWG), which sponsored the workshop that led ultimately to this publication, is an open organization with a deliberate process for developing community technical standards. The National Evolutionary Synthesis Center (NESCent) has supported various projects focused on interoperability, including an Evolutionary Informatics working group and several hackathons. NeXML (Figure 2) is being developed using an open-source open-development model. Anyone can join the mailing list, and those who make useful contributions are invited to join the developer team. TreeBASE has become an open-source project with a public mailing list for discussion and bug reports. The MIAPA project, an informal consortium that communicates via a public mailing list (Table 1), is an open consortium that continues to gather new members— for instance, all the authors of the present article participate, but none of us were authors of the original MIAPA article [15].

Indeed, the evolutionary research community appears poised to experience dramatic increases in integration and synthesis mediated by sharing of data. Phylogenies are used widely in biological and biomedical research. Evolutionary researchers are increasingly aware of the enormous potential for data integration and synthesis [4,6]. Recognizing this potential, professional societies and journal editorial boards recently embraced mandatory archiving [10]. Software developers in this field traditionally have taken a broad interdisciplinary view of data models [13], and have shown a willingness to adopt or adapt existing standards, rather than invent new formats, all of which increase the potential for integration and synthesis. To make good on its potential, evolutionary informatics now requires broad engagement of stakeholders to develop a common understanding of the standards, technologies, and practices that facilitate sharing and re-use of data.

## Availability of supporting data

Supporting data (http://dx.doi.org/10.5061/dryad.h6pf365t) have been deposited in the Dryad Repository, including: (1) a README file describing the contents of each file, (2) user stories regarding barriers to re-use, and (3) spreadsheets (in Excel and CSV format) with the results of the several literature surveys described here.

## Competing interests

The authors declare that they have no competing financial interests. The authors participate in several projects or groups mentioned in the text. RV is the leader of the NeXML project. SK co-directs the TimeTree project. AS is a contributor to NeXML and the instigator of the miapa-discuss email list. All authors are members of the miapa-discuss email list, and the email list of the TDWG Phylogenetic Standards Interest Group, from which this project emerged.

## Authors’ contributions

AS and DR conceived the initial idea; AS, JW, DR and RV evaluated relevant file formats, archives and policies; BO, AS, and RM carried out the systematic analysis of archiving and re-use in samples of publications; ELG analyzed re-use of leaf economics spectrum data; AS, RM, SK, RV and ELG gathered and analyzed user stories; AS and JW wrote an initial report of findings, expanded by AS into a draft manuscript; AS, SK, BO and RV revised the manuscript; RM, AS and RV prepared the supporting data package. All authors read and approved the final manuscript.
